# Construction of Ultrastable Conjugated Microporous Polymers Containing Thiophene and Fluorene for Metal Ion Sensing and Energy Storage

**DOI:** 10.3390/mi13091466

**Published:** 2022-09-04

**Authors:** Mohamed Gamal Mohamed, Huan-Yu Hu, Manivannan Madhu, Mohsin Ejaz, Santosh U. Sharma, Wei-Lung Tseng, Maha Mohamed Samy, Cheng-Wei Huang, Jyh-Tsung Lee, Shiao-Wei Kuo

**Affiliations:** 1 Department of Materials and Optoelectronic Science, College of Semiconductor and Advanced Technology Research, Center for Functional Polymers and Supramolecular Materials, National Sun Yat-sen University, Kaohsiung 804, Taiwan; 2 Chemistry Department, Faculty of Science, Assiut University, Assiut 71515, Egypt; 3 Department of Chemistry, National Sun Yat-sen University, Kaohsiung 804, Taiwan; 4 Department of Chemical and Materials Engineering, National Kaohsiung University of Science and Technology, Kaohsiung 807, Taiwan; 5 Department of Medicinal and Applied Chemistry, Kaohsiung Medical University, Kaohsiung 807, Taiwan

**Keywords:** Suzuki coupling reaction, thiophene, fluorene, conjugated microporous polymers, supercapacitor

## Abstract

In this study, we have used the one-pot polycondensation method to prepare novel 2D conjugated microporous polymers (Th-F-CMP) containing thiophene (Th) and fluorene (Fl) moieties through the Suzuki cross-coupling reaction. The thermogravimetric analysis (TGA) data revealed that Th-F-CMP (*T_d10_* = 418 °C, char yield: 53 wt%). Based on BET analyses, the Th-F-CMP sample displayed a BET specific surface area of 30 m^2^ g^−1^, and the pore size was 2.61 nm. Next, to show the effectiveness of our study, we utilized Th-F-CMP as a fluorescence probe for the selective detection of Fe^3+^ ions at neutral pH with a linear range from 2.0 to 25.0 nM (*R*^2^ = 0.9349). Furthermore, the electrochemical experimental studies showed that the Th-F-CMP framework had a superior specific capacity of 84.7 F g^−1^ at a current density of 0.5 A g^−1^ and outstanding capacitance retention (88%) over 2000 cycles.

## 1. Introduction

**Figure 1:**
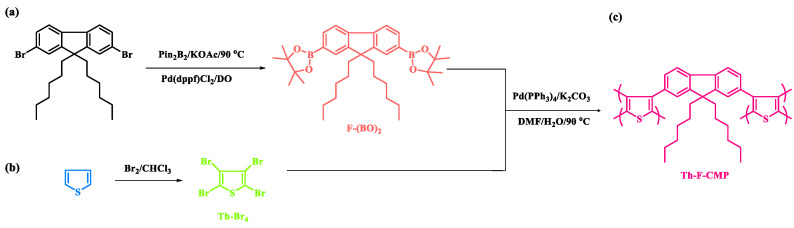
Schematic scheme for the preparation of (**a**) F-(BO)_2_, (**b**) Th-Br_4_, and (**c**) Th-F-CMP.

Energy is a necessity for the existence and advancement of human civilization. The need for traditional fossil fuels is increasing in line with the rapid development of society, the economy, and the enormous rise in environmental pollution [1,2,3,4,5,6]. Electricity can be produced from clean, renewable sources like solar, tidal, or wind energy as power sources, and it has a lot of potential to meet our long-term energy demands. However, dependence on these renewable resources is not a smart idea because they only supply energy when the raw ingredients are running out. In addition, the acceleration of climate change indicates that fossil fuels also impact the environment [7,8]. Thus, designing efficient solutions to store energy and resolve the current situation is essential. Supercapacitors are often used in electrical and electronic appliances because they are effective at storing energy and have a high-power density, cycle stability, energy density, cycling life, and charge/discharge rate [9,10]. Supercapacitors’ key benefit is their excellent stability, resulting from their charge-storing mechanism. Since it is reversible, the charging-discharging cycle does not change the electrode volume [11]. The characteristics of the electrode materials have a significant impact on supercapacitor performance [9]. Hydroxides, metal oxides, and carbon-based materials have been used as electrodes for supercapacitors [12,13,14,15]. Inorganic materials for the electrodes have a negative impact on the environment, so organic electrode materials have emerged as a possible substitute for supercapacitors [16,17]. The main shortcoming of a supercapacitor is its limiting operating window [18]. The electrolyte decomposes when too much voltage is supplied [19]. However, supercapacitors have drawbacks although these limitations can be solved by contributing to advancements in polymer research. Porous organic polymers (POPs) have piqued the curiosity of researchers as a promising material in recent years. It offers the advantages of high specific surface area, variable pore size, and a lower density than inorganic materials. Recently, they have been used in a variety of applications such as hydrogen evolution, sensing, energy storage, water treatment, gas separation, and optoelectronics [20,21,22,23,24,25,26,27,28,29,30,31,32,33]. Conjugated microporous polymers (CMPs) are porous organic polymers with π-conjugation in their microporous structures. Therefore, such materials are being prepared with various structures and characteristics due to the continuous accessibility of building blocks and reactions. [34,35,36,37]. In addition, they also have a vast pore structure, which can assist them as organic electrode materials for supercapacitors. CMPs have exceptional electrochemical performance as they have a particular π -conjugated structure with redox activity [38,39,40,41,42,43]. In addition, for energy storage applications, CMPs having fluorophores in their structure promote exciton migration across the network, allowing luminous capabilities [44]. Compared to typical conjugated polymers, the unique micropores will restrict chain aggregation, suppressing excitation energy dissipation and consequently improving light-emitting capabilities [44,45,46,47,48]. CMPs with strong luminescent characteristics and visible emission colors may be suitable choices as sensing materials [49,50,51,52,53]. Thus, using porous polymers containing thiophene (Th) and fluorene (F) molecules as active materials in optoelectronic devices, photocatalytic applications, and chemical sensing have been widely discovered due to their facile preparation and high quantum yield [54,55,56]. In this work, we have inserted Th and F as building blocks to develop a novel CMP (named Th-F-CMP) through a one-pot polycondensation method using the Suzuki−Miyaura coupling reaction, as presented in Figure 1. All properties, including chemical structures, morphology, crystalline, thermal degradation, char yield, and porosity of Th-F-CMP, were carefully studied and examined using different instruments, as shown in this study. In addition, the Th-F-CMP possesses excellent thermal stability (*T_d10_* = 410 °C with a carbon residue of 53 wt% at 800 °C). Based on three electrode measurements, the analyses reveal the capability of Th-F-CMP for real and hybrid electric energy storage applications.

## 2. Materials and Methods

### 2.1. Materials

Bromine (Br_2_), sodium thiosulfate, thiophene (Th), 2,7-dibromo-9,9-dihexylfluorene (F-DH-Br_2_), bis(pinacolato), diborane (pin_2_B_2_), 1,4-dioxane (DO), potassium carbonate (K_2_CO_3_, 99.9%), anhydrous magnesium sulfate (MgSO_4_, 99.5%), tetrahydrofuran (THF), acetone, methanol (MeOH), and chloroform (CHCl_3_) were purchased from Alfa Aesar. Pd(dppf)Cl_2_ and Pd(PPh_3_)_4_ were ordered from Sigma–Aldrich.

### 2.2. Synthesis of 2,7-Bis(4,4,5,5-tetramethyl-1,3,2-dioxaborolan-2-yl)-9,9-dihexylfluorene [F-(BO)_2_]

**Figure 2:**
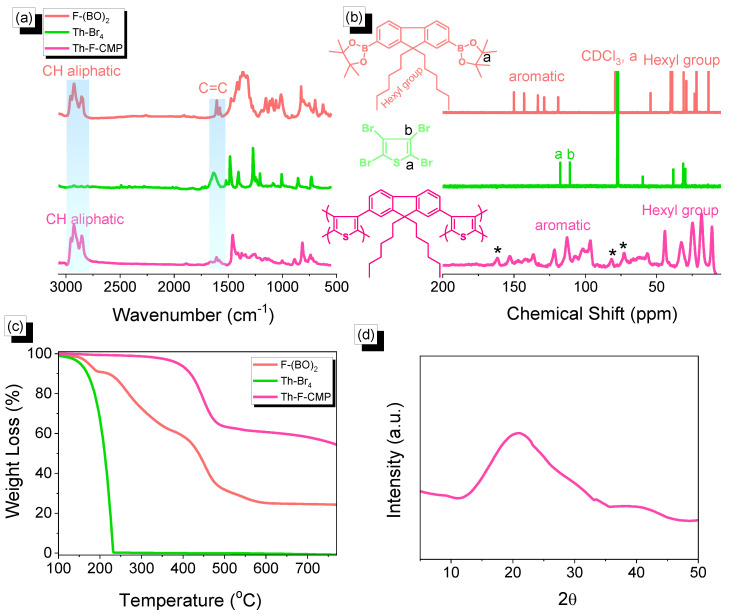
(**a**) FTIR, (**b**) ^13^C NMR (**c**) TGA analyses of F-(BO)_2_, Th-Br_4_, Th-F-CMP, and (**d**) Th-F-CMP XRD profile. * is the side band of solid state nuclear magnetic resonance spectroscopy (NMR).

In a round-bottom flask 250 mL, F-DH-Br_2_ (1 g, 2 mmol), KOAc (2.25 g, 12 mmol), pin_2_B_2_ (1 g, 4 mmol), and Pd(dppf)Cl_2_ (0.12 g, 0.014 mmol) were mixed with DO under N_2_ and stirred for 48 h at 90 °C. When the mixture cooled down to room temperature, water and chloroform were added to the mixture. The organic layer was washed with brine and water and dried over anhydrous MgSO_4_. Finally, the obtained powder was purified by silica gel column chromatography (the eluent used was petroleum) to collect a white solid. FTIR (KBr, cm^–1^, Figure 2a): 2933, 2843, 1610 (C=C). ^1^H NMR (500 MHz, CDCl_3_, δ, ppm, Figure S1): 7.70–7.81, 1.4, 1.01–1.11, and 0.72–0.76. ^13^C NMR (125 MHz, CDCl_3_, δ, ppm, Figure 2b): 150, 142, 133, 128, 119, 54, 39, 31, 29, 23, 22.

### 2.3. Synthesis of 2,3,4,5-Tetrabromothiophene (Th-Br_4_)

Br_2_ (5.3 mL, 0.033 mmol) and Th (2.0 g, 0.023 mmol) were dissolved in CHCl_3_ (30 mL) and then the mixture was refluxed for 24 hours at 0 ℃. After cooling, the solution mixture was added to cool, saturated sodium thiosulfate. The obtained solid was purified in hot ethanol to remove any impurities from the monomer to obtain Th-Br_4_ as a white powder (Figure 1, 80%, T_m_: 119 °C). FTIR (KBr, cm^–1^, Figure 2a): 1636 (C=C), 852 (C–S). ^1^H NMR (500 MHz, DMSO, δ, ppm, Figure S2): No peak was detected. ^13^C NMR (125 MHz, CDCl_3_, δ, ppm, Figure 2b): 116.936, 110.284.

### 2.4. Synthesis of Th-F-CMP

Th-Br_4_ (100 mg, 0.25 mmol), F-(BO)_2_ (294 mg, 0.50 mmol), K_2_CO_3_ (280 mg, 2.0 mmol), and Pd (30.0 mg, 0.025 mmol) in DMF (8 mL)/H_2_O (2 mL) were mixed to a 25 mL two-necked flask, and heated under N_2_ at 90 °C for 3 days with stirring. After cooling to room temperature, the solid precipitate was filtered and washed well by THF, MeOH, and acetone. The solid was dried in an oven under a vacuum at 100 °C overnight to obtain a green solid (0.19 g, 73%, Figure 1b). FTIR (KBr, cm^–1^, Figure 2a): 2930, 2850, and 1603 (C=C).

### 2.5. Procedures for Sensing Fe^3+^ Ions

To sense the targeted analytes, 250 μL of Th-F-CMP (0.05 mg/mL) which was readily available in water suspension obtained via ultrasonication method, were added to 1 mL centrifuge tubes containing 200 μL of PBS solution (pH = 7.0, 0.1 M). Then, different concentrations of Fe^3+^ ions (150 μL; 0.002–200 µM) were added, followed by incubating them at room temperature with continuous and constant stirring under a vortex. After an hour, 500 μL of the resultant solution was pipetted out and transferred into a 1 mL quartz cuvette. The fluorescence spectra were recorded by operating the fluorescence spectrophotometer at the excitation wavelength of 380 nm.

### 2.6. Selectivity and Interference Tests

To study the effectiveness of Th-F-CMP in sensing Fe^3+^ species, the selectivity of the Th-F-CMP was monitored at fixed optical parameters such as the excitation wavelength of 380 nm and its corresponding emission wavelength of 477 nm. To perform this, the same procedure used for sensing in the above case was followed simply by substituting various other kinds of metal ions, such as Pb^2+^ and Fe^2+^, Zn^2+^, Co^2+^, Mn^2+^, Cr^3+^, Cu^2+^, Hg^+^, Ni^2+^, and Ag^+^ [500 µM] in place of Fe^3+^.

### 2.7. Electrochemical Characterization

The electrochemical experiments were performed in a three-electrode cell using an Autolab potentiostat (PGSTAT204) and 1 M KOH as the aqueous electrolyte. The GCE was used as the working electrode (diameter: 5.61 mm; 0.2475 cm^2^); a Pt wire was used as the counter electrode; and Hg/HgO (RE-1B, BAS) was the reference electrode. All reported potentials refer to the Hg/HgO potential. A slurry was prepared by dispersing Th-F-CMP (45 wt%), carbon black (45 wt%), and Nafion (10 wt%) in a mixture of (EtOH/H_2_O) (200 µL: 800 µL) and then sonicated for 1 h. A portion of this slurry (10 µL) was pipetted onto the tip of the electrode, which was then dried in air for 30 min before use. The electrochemical performance was studied through CV at various sweep rates (5–200 mV s^–1^) and through the GCD method in the potential range from −1.0 V to 0.0 V (vs. Hg/HgO) at various current densities (0.5–20 A g^–1^) in 1 M KOH as the aqueous electrolyte solution.

## 3. Results

### 3.1. Synthesis and Characterization of F-(BO)_2_, Th-Br_4_, and Th-F-CMP

**Figure 3:**
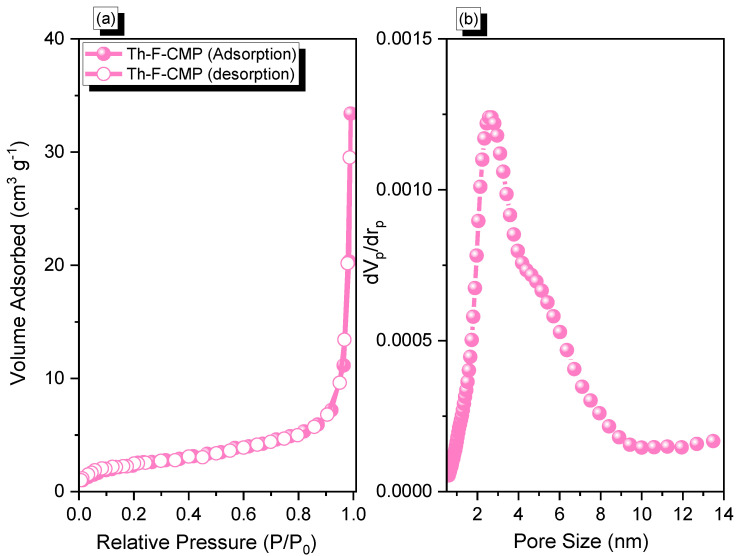
(**a**) N_2_ adsorption-desorption and (**b**) pore size distribution of Th-F-CMP.

The Th-F-CMP framework was prepared through three steps, as shown in Figure 1. First, The F-(BO)_2_ was easily synthesized with a high yield by reacting F-DH-Br_2_ with KOAc, pin_2_B_2_, and Pd(dppf)Cl_2_ for 48 h under reflux (Figure 1a). Secondly, the Th-Br_4_ was easily prepared through the reaction of the Th with Br_2_ solution in CHCl_3_ as the solvent at 0 ℃ (Figure 1b). Finally, our Th-F-CMP was synthesized to a green solid at a high yield by the Suzuki coupling reaction of F-(BO)_2_ with Th-Br_4_ in a mixture of DMF and H_2_O with K_2_CO_3_ and Pd(PPh_3_)_4_ for 72 h under reflux (Figure 1c). The Th-F-CMP sample is not soluble and undecomposed in all organic solvents (MeOH, ETOH, H_2_O, DMF, DMSO, DCM, CHCl_3_, and THF), indicating the attachment of both fluorene and thiophene moieties through Suzuki reaction and forming Th-F-CMP with high chemical stability, degree of polymerization, and high crosslinking density. The protons signals in the 1H-NMR spectrum of F-(BO)_2_ (Figure S1) were 7.70–7.81, 1.4, 1.01–1.11, and 0.72–0.76 ppm, corresponding to the aromatic ring, C(CH_3_)_2_, CH_2_, and CH_3_ groups, respectively. While the ^1^H-NMR spectrum of Th-Br_2_ does not show any proton signals (Figure S2). Furthermore, as shown in Figure 2, the chemical structures and thermal stability of the resulting F-(BO)_2_, Th-Br_4,_ and Th-F-CMP were investigated using FTIR, ^13^C NMR solid, and TGA analyses. In their FTIR spectra, all three F-(BO)_2_, Th-Br_4_, and Th-F-CMP showed main absorption characteristics peaks in the range 3055–3030 cm^−1^ and 1630–1610 cm^−1^, corresponding to the stretching vibration of C−H aromatic and C=C units, respectively (Figure 2a). The stretching vibration of CH aliphatic absorption bands appeared in the range of 2930–2850 cm^−1^ in both F-(BO)_2_ and Th-F-CMP (Figure 2a). In addition, the signals of aromatic carbon nuclei in the ^13^C NMR spectra (Figure 2b) were centered at 149.93–119.25 ppm and 152.66–96.85 ppm for F-(BO)_2_ and Th-F-CMP, respectively. The two signals of the aromatic carbons for the thiophene unit in the Th-Br_4_ compound were found at 117.65 and 111.06 ppm (Figure 2b). The successful synthesis of F-(BO)_2_ was confirmed by the carbon signal at 79.35 ppm for the C(CH_3_)_2_ unit. Furthermore, signals of aliphatic carbon nuclei were found at 55.16–14.12 ppm and 56.85–11.39 ppm for hexyl groups in both F-(BO)_2_ and Th-F-CMP structures. To better understand the thermal stability properties of F-(BO)_2_, Th-Br_4_, and Th-F-CMP during thermogravimetric analyses (TGA) all three CMPs were heated from 40 to 800 ℃ under a N_2_ atmosphere (Figure 2c). As observed in the TGA data, the 10% weight losses of F-(BO)_2_, Th-Br_4,_ and Th-F-CMP under N_2_ were observed at 220, 170, and 418 ℃, respectively. The char yields at 800 °C for F-(BO)_2_, Th-Br_4,_ and Th-F-CMP were 25, 0, and 53 wt%, respectively. Based on TGA data, the Th-F-CMP sample exhibited excellent thermal stability properties with high char yield, which is considered an important phenomenon in their real applications. As shown above, NMR, FTIR, and TGA data confirmed the successful synthesis of the Th-F-CMP sample. The appearance of two broad peaks at 20.95 and 40.16º in the powder X-ray diffraction (PXRD) profile (Figure 2d), indicates that Th-F-CMP has an amorphous structure. Furthermore, the porosity properties of Th-F-CMP material were determined at 77 K using nitrogen adsorption-desorption measurements (Figure 3).

The Th-F-CMP material's N_2_ adsorption profile (Figure 3a) showed a rapid increase in N_2_ capture when P/P0 > 0.8, indicating the Th-F-CMP framework structure includes mesoporous and microporous characteristics. In addition, the N_2_ isotherm profile of the Th-F-CMP displayed Type III (according to IUPAC classification). The Brunauer-Emmett-Teller (BET) surface area and total pore volume of the Th-F-CMP were found to be 30 m^2^ g^−1^ and 0.05 cm^3^ g^−1^, respectively. As seen, our Th-F-CMP had a low specific surface area, presumably due to the long hexyl group and flexible structure of the fluorene unit relative to those of the other rigid building blocks used for the synthesis of POPs. Non-local density functional theory (NLDFT) was applied to investigate the pore size diameter of the Th-F-CMP. We found that the pore size of the Th-F-CMP was 2.61 nm (Figure 3b).

**Figure 4:**
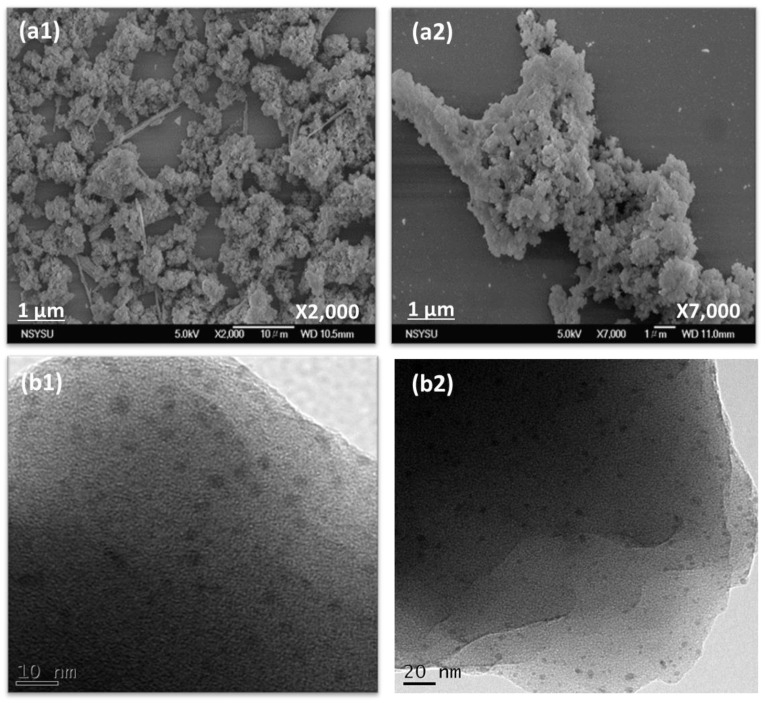
SEM (**a1**,**a2**) and TEM (**b1**,**b2**) images of Th-F-CMP.

The morphologies of the porous Th-F-CMP were investigated by field emission scanning electron microscopy (FE-SEM) and high-resolution transmission electron microscopy (HR-TEM), respectively (Figure 4). As observed, SEM images depicted that Th-F-CMP contained irregularly sand aggregated spheres of nanoparticles (Figure 4a1,a2). In contrast, HR-TEM images (Figure 4b1,b2) illustrated that Th-F-CMP is composed of a porous network structure.

### 3.2. Metal Ions Detection via Th-F-CMP Probe

**Figure 5:**
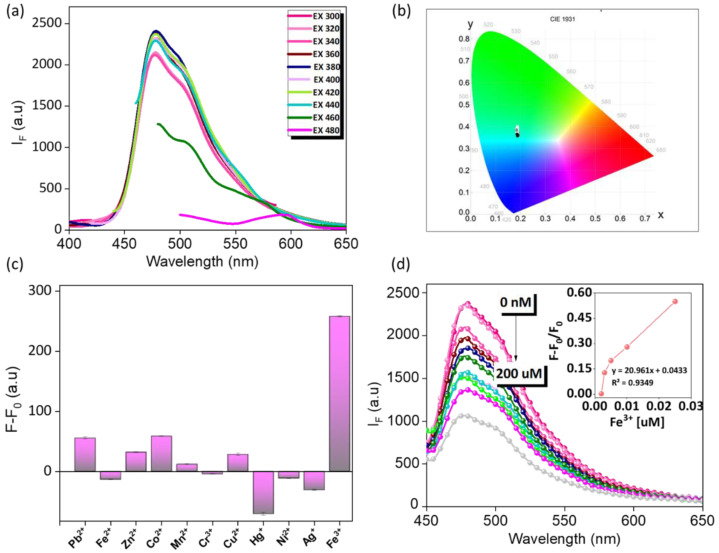
(**a**,**b**) optical characterization and excitation independent fluorescence spectrum (**a**) and CIE color chromaticity (**b**). (**c**,**d**) sensing system. Selectivity studies of the proposed probe towards Fe^3+^ (**c**) and quantification of Fe^3+^ ion with different concentration (**d**), and their linear calibration curve inserts (**d**) measured at pH 7.

The prepared Th-F-CMP powder was taken in a glass tube and suspended in ultrapure DI water via sonication treatment. Finally, at the end of 25 min, the partially dispersed transparent solution was carefully separated and stored in a separate glass tube for further experimental analysis. To investigate the optical properties of the prepared THF-CMP, 500 μL of the above collected fine solution was taken, and its emission spectrum was recorded using a fluorescence spectrophotometer. As shown in Figure 5a, the Th-F-CMP shows strong fluorescence intensity at 477 nm. Moreover, the obtained emission was highly independent of the seriousness of excitation starting from excitation 300 to 400 nm, which is quite the opposite to other kinds of fluorescence and phosphorescence-based reported probes that mislead them to multiple or white emitting fluorescence [57]. Our prepared probe shows a strong cyan fluorescence color due to its independent excitation characteristics. A multiple point in the CIE color chart shown in (Figure 5b) indicates that our Th-F-CMP emits strong cyan fluorescence with similar kinds of CIE (X, Y) coordinates such as 0.189, 0.375; 0.189, 0.371; 0.188, 0.367; 0.188, 0.362; 0.190, 0.360; 0.189, 0.359; 0.188, 0.357; 0.188, 0.357; 0.186; and 0.365 throughout the excitation region of 300–440 nm respectively. Next, we evaluated the effect of pH on our prepared Th-F-CMP and found that the pH solution in acidic, neutral, and basic regions rarely influences the emission intensity (Figure S3), which highly motivates us to develop a sensor for Fe^3+^ metal ion. As shown in (Figure 5c), the fluorescence intensity (F − F_0_) of the proposed probe was shown to be higher than that of other related metal ion species, while the concentrations of all metal ions remain the same. In addition, the selectivity of the proposed probe for Fe^3+^ was also evolved in the presence of various other metal ions (Figure S4). Here, the notations F and F_0_ represent the fluorescence intensity of the proposed probe in the absence and presence of various metal ions measured at pH 7. Therefore, considering the high selectivity of the probe toward Fe^3+^, we next determined the different concentrations of Fe^3+^. As shown in (Figure 5d), increasing the concentration of Fe^3+^ resulted in a continuous decrease in the emission intensity recorded at 477 nm, which could be attributed to π-π interaction between the aromatic structures of the synthesized probe and Fe^3+^ ion. Additionally, the energy transfer and absorption competition quenching mechanism between Th-F-CMP and Fe^3+^ leads to fluorescence quenching. Plotting the value of (F − F_0_)/F_0_ versus the concentrations of Fe^3+^ gives a linear calibration curve (inset in Figure 5d). The correlation coefficients (*R*^2^) were 0.9349 for determining Fe^3+^ over the concentration ranges of 0.001–0.01 µM.

### 3.3. Electrochemical Performance of Th-F-CMP

**Figure 6:**
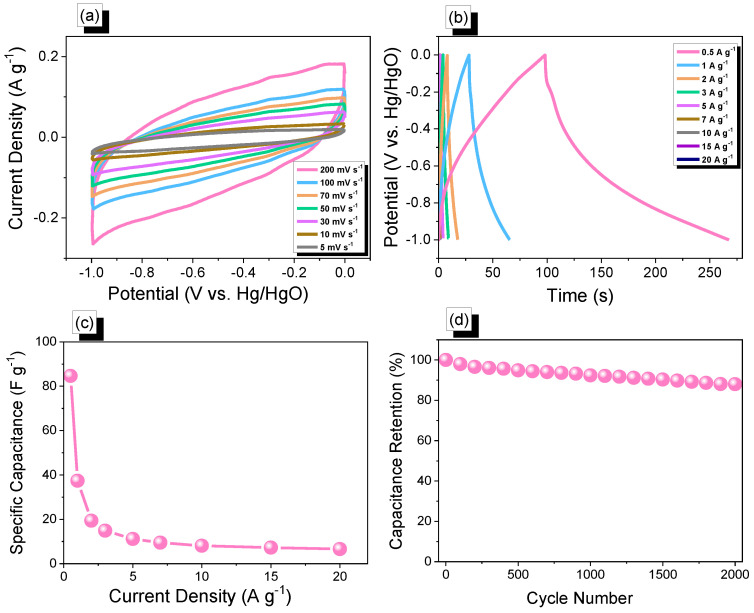
(**a**) Cyclic voltammetry, (**b**) GCD curves, (**c**) specific capacity vs. current density curve, and (**d**) capacity retention curve of Th-F-CMP.

**Figure 7:**
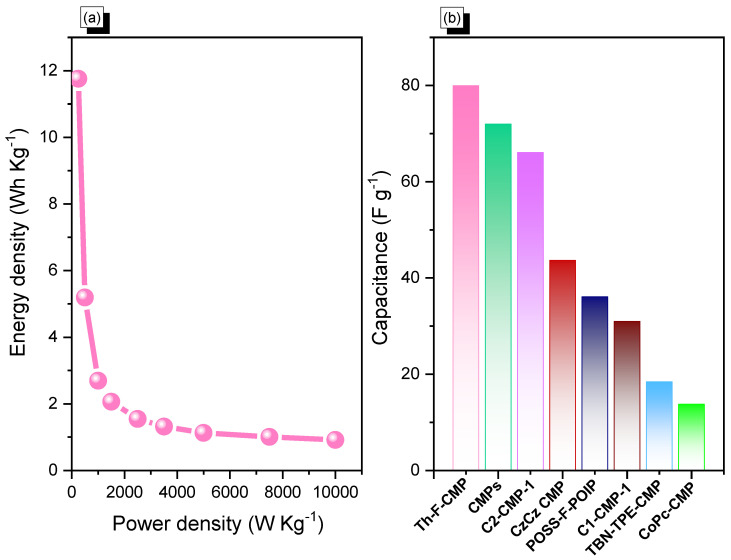
(**a**) Ragone plot and (**b**) comparison of the electrochemical performance of the Th-F-CMP material with other CMPs candidates.

In addition, cyclic voltammetry and galvanostatic charge-discharge measurements were performed with glassy carbon electrode, mercury electrode, and platinum electrode as working, reference, and counter electrodes, respectively, in the aqueous electrolyte of 1.0 M KOH within a potential range from 0 to −1.0 V, to assess the electrochemical performances of Th-F-CMP, conjugated microporous polymers for their viability as electrode materials for energy storage applications. The related cyclic voltammetry curves of the Th-F-CMP at various scan speeds between 5 and 200 mV s^−1^ are shown in (Figure 6a). The compound persisted with scan sweeping and generated the distinctive rectangle-like humped shape, proving that the as-obtained compound is robust in terms of current sweep and indicates capacitance via the EDLC mechanism. As current density rises and scan rates increase, the CV curve of Th-F-CMP has been changed, exhibiting improved stability, rate efficiency, and kinetics due to its porous structure and spherical particle structure. The GCD curves for Th-F-CMP at various current densities between 0.5 and 20 A g^−1^ are shown in (Figure 6b). This CMP’s GCD curves were triangle-shaped with slight bends that showed both EDLC and pseudocapacitive behavior. Moreover, the fact that the discharging time for this Th-F-CMP was more significant than the charging time shows that this material has a larger capacity. The GCD curves of Th-F-CMP were used to determine the specific capacitance, which was found to be 84.7, 37.4, 19.4, 14.87, 11.15, 9.5, 8.13, 7.25, and 6.6 F g^−1^ at 0.5, 1, 2, 3, 5, 7, 10, 15, and 20 A g^−1^, respectively, as shown in (Figure 6c). Furthermore, (Figure 6d) examined the extended cycling stability of Th-F-CMP electrode material for 2000 charge-discharge cycles at a higher current density of 10 A g^−1^. As we can see, Th-F-CMP had a high-capacity retention determined to be 88.00%. Thus, the superior performance of Th-F-CMP compound as an electrode material for energy storage revealed its potential for reliable energy storage compounds with a porous nature. Previously, Mohamed et al. reported an ultrastable porous POSS-A-POIP with the highest specific surface area, delivering a specific capacitance of 36.2 F g^−1^ at a current density of 0.5 A g^−1^ [58]. In addition, Khattak and co-workers have studied the potential of these redox-active conjugated microporous polymers for energy storage with graphene oxide [59]. These CMPs have exhibited a higher surface area of more than 600 m^2^ g^−1^ with a higher specific capacitance of 67.38 F g^−1^ at a low current density of 0.5 A g^−1^ [59]. The Ragone plot (Figure 7a) revealed that the energy density value for Th-F-CMP in the three electrodes system was 11.83 Wh kg^–1^, respectively. Our Th-F-CMP material displayed excellent electrochemical performance compared with other porous materials, as presented in (Figure 7b). Hence, based on the previously reported literature on CMPs for energy storage (Figure 7b), Th-F-CMP has exhibited extraordinary performance as a potential candidate for an energy storage system as an ideal electrode material.

## 4. Conclusions

We designed and constructed a novel CMP (Th-F-CMP) containing Th and F units through the Suzuki cross-coupling reaction. TGA data demonstrated that Th-F-CMP had a high T*_d10_* around 418 °C, with a char yield of 53 wt%. In addition, the Th- F-CMP displayed a specific surface area of 30 m^2^ g^−1^ and a pore volume of 0.05 cm^3^ g^−1^. Furthermore, we used the fluorescence spectrophotometer to confirm the prepared probe Th-F-CMP as a fluorescence probe and is capable of the sensitive and selective determination of Fe^3+^ neutral pH. Finally, we have evaluated the electrochemical performance of the three-electrodes for real application. The Th-F-CMP has delivered an outstanding specific capacity of 84.7 F g^−1^ at a current density of 0.5 A g^−1^. In addition, the Th-F-CMP has exhibited superior cycling stability for 2000 charge-discharge cycles at a higher current density of 10 A g^−1^ with a capacitance retention of over 88%. We expect our newly obtained CMP material to be an excellent candidate for energy storage.

## Data Availability

Data will be available from the first author on demand.

## Supplementary Materials

The following supporting information can be downloaded at: https://www.mdpi.com/article/10.3390/mi13091466/s1, Figure S1: ^1^H NMR spectrum of F-(BO)_2_; Figure S2: ^1^H NMR spectrum of Th-Br_4_; Figure S3: Effect of pH solution on the emission intensity of the Th-F-CMP probe; Figure S4: The selectivity of the proposed probe for Fe^3+^ was also evolved in the presence of various other metal ions.
