# Supplementary material for: Construction of Ultrastable Conjugated Microporous Polymers Containing Thiophene and Fluorene for Metal Ion Sensing and Energy Storage
Source: Micromachines (Basel). 2022 Sep 4;13(9):1466. doi: 10.3390/mi13091466 (PMC9505267; doi:10.3390/mi13091466)
Supplement: Supplementary file 1 [file micromachines-13-01466-s001.zip › micromachines-1903186-supplementary.pdf]

Supporting Information

Construction of Ultrastable Conjugated Microporous Polymers Containing Thiophene and Fluorene for Metal Ion Sensing and Energy Storage

Mohamed Gamal Mohamed[1,2*], Huan-Yu Hu[1], Manivannan Madhu[3], Mohsin Ejaz[1], Santosh U Sharma[3], Wei-Lung Tseng[3], Maha Mohamed Samy[1,2], Cheng-Wei Huang[4], Jyh-Tsung Lee[3], and Shiao-Wei Kuo[1,5*]

[1] Department of Materials and Optoelectronic Science, College of Semiconductor and Advanced Technology R search, Center for Functional Polymers and Supramolecular Materials, National Sun Yat-Sen University, Kaohsiung 804, Taiwan

[2]Chemistry Department, Faculty of Science, Assiut University, Assiut, Egypt

[3]Department of Chemistry, National Sun Yat-Sen University, Kaohsiung 80424, Taiwan

[4] Department of Chemical and Materials Engineering, National Kaohsiung University of Science and Technology, Kaohsiung, 80778, Taiwan

[5] Department of Medicinal and Applied Chemistry, Kaohsiung Medical University, Kaohsiung 807, Taiwan

* Corresponding authors: E-mail: mgamal.eldin34@gmail.com (M. G. Mohamed), and (kuosw@faculty.nsysu.edu.tw (S. W. Kuo)

Characterization

FTIR spectra were collected on a Bruker Tensor 27 FTIR spectrophotometer with a resolution of 4 cm^{-1} by using KBr disk method. ^{13}C nuclear magnetic resonance (NMR) spectra were examined by using an INOVA 500 instrument with DMSO as the solvent and TMS as the external standard. Chemical shifts are reported in parts per million (ppm). The curing behavior and thermal stabilities of the samples were performed by using a TG Q-50 thermogravimetric analyzer under an N_2 atmosphere; the cured sample (ca. 5 mg) was put in a Pt cell with a heating rate of 20 °C min^{-1} from 100 to 800 °C under an N_2 flow rate of 60 mL min^{-1}. Wide-angle X-ray diffraction (WAXD) patterns were measured by the wiggler beamline BL17A1 of the National Synchrotron Radiation Research Center (NSRRC), Taiwan. A triangular bent Si (111) single crystal was used to get a monochromated beam having a wavelength (λ) of 1.33 Å. The morphologies of the polymer samples were examined by Field emission scanning electron microscopy (FE-SEM; JEOL JSM7610F) and also by transmission electron microscope (TEM) using a JEOL-2100 instrument at an accelerating voltage of 200 kV. BET surface area and porosimetry measurements of samples (ca. 40–100 mg) were measured using BEL Master^{TM}/BEL sim^{TM} (v. 3.0.0). N_2 adsorption and desorption isotherms were generated through incremental exposure to ultrahigh-purity N_2 (up to ca. 1 atm) in a liquid N_2 (77 K) bath. Surface parameters were calculated using BET adsorption models in the instrument's software. The pore size of the prepared samples was determined by using nonlocal density functional theory (NLDFT). Fluorescence emission spectrum and color chromaticity were recorded on a Hitachi F-7000 fluorometer (Hitachi, Tokyo, Japan), and an Edinburgh FS5 spectrofluorometer (Edinburgh Instruments Ltd., UK).

Electrochemical Analysis

Working Electrode Cleaning: Before use, the glassy carbon electrode (GCE) was polished several times with 0.05-μm alumina powder, washed with EtOH after each polishing step, and cleaned through sonication (5 min) in a water bath, washed with EtOH, and then dried in air.

Electrochemical Characterization: The electrochemical experiments were performed in a three-electrode cell using an Autolab potentiostat (PGSTAT204) and 1 M KOH as the aqueous electrolyte. The GCE was used as the working electrode (diameter: 5.61 mm; 0.2475 cm^2); a Pt wire was used as the counter electrode; Hg/HgO (RE-1B, BAS) was the reference electrode. All reported potentials refer to the Hg/HgO potential. A slurry was prepared by dispersing Th-F-CMP (45 wt %), carbon black (45 wt %), and Nafion (10 wt %) in a mixture of (EtOH/ H_2O) (200 μL: 800 μL) and then sonicated for 1 h. A portion of this slurry (10 μL) was pipetted onto the tip of the electrode, which was then dried in air for 30 min before use. The electrochemical performance was studied through CV at various sweep rates (5–200 mV s^{-1}) and through the GCD method in the potential range from -1.0 V and 0.0 V (vs. Hg/HgO) at various current densities (0.5–20 A g^{-1}) in 1 M KOH as the aqueous electrolyte solution.

The specific capacitance was calculated from the GCD data using the equation.

$$C_s = (I\Delta t)/(m\Delta V) \qquad \textbf{(S1)}$$

Where C_s (F g^{-1}) is the specific capacitance of the supercapacitor, I (A) is the discharge current, ΔV (V) is the potential window, Δt (s) is the discharge time, and m (g) is the mass of the NPC on the electrode. The energy density (E, W h kg^{-1}) and power density (P, W kg^{-1}) were calculated using the equations.

$$E = 1000C(\Delta V)^2/(2 \times 3600) \quad (\mathbf{S2})$$

$$P = E/(t/3600) \quad (\mathbf{S3})$$

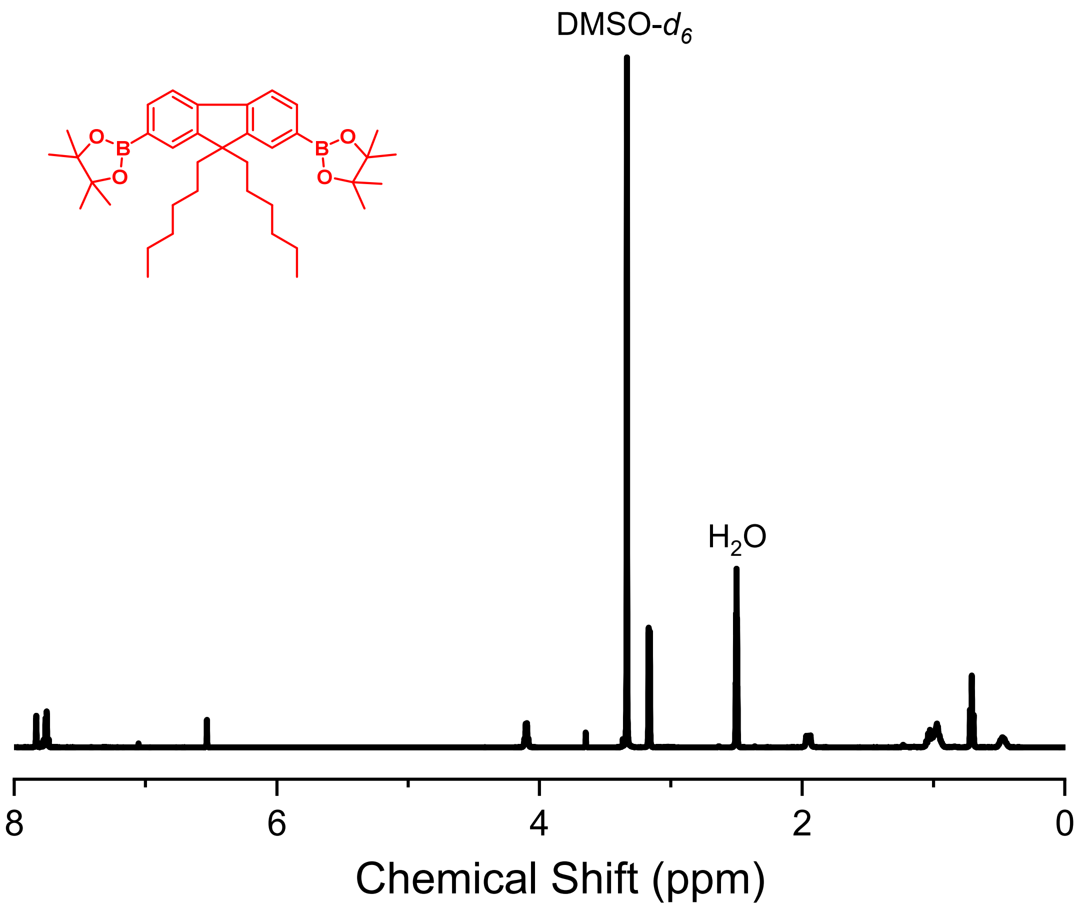

Figure S1. ^1H NMR spectrum of F-(BO)2.

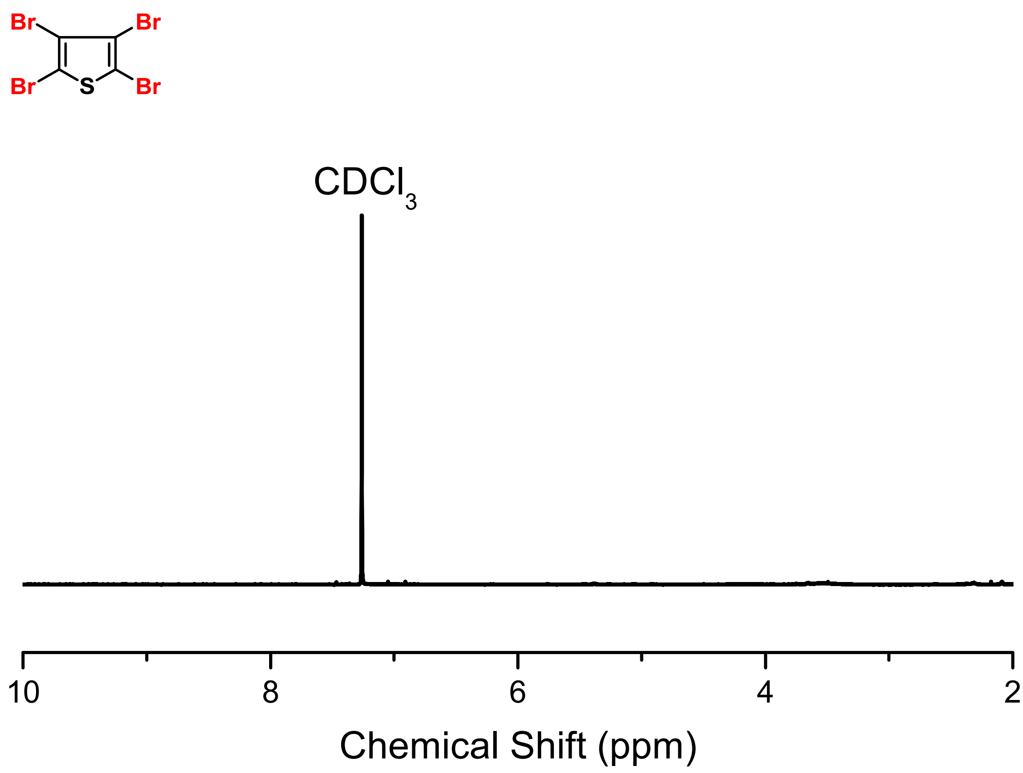

Figure S2. ^{1}H NMR spectrum of Th-Br4.

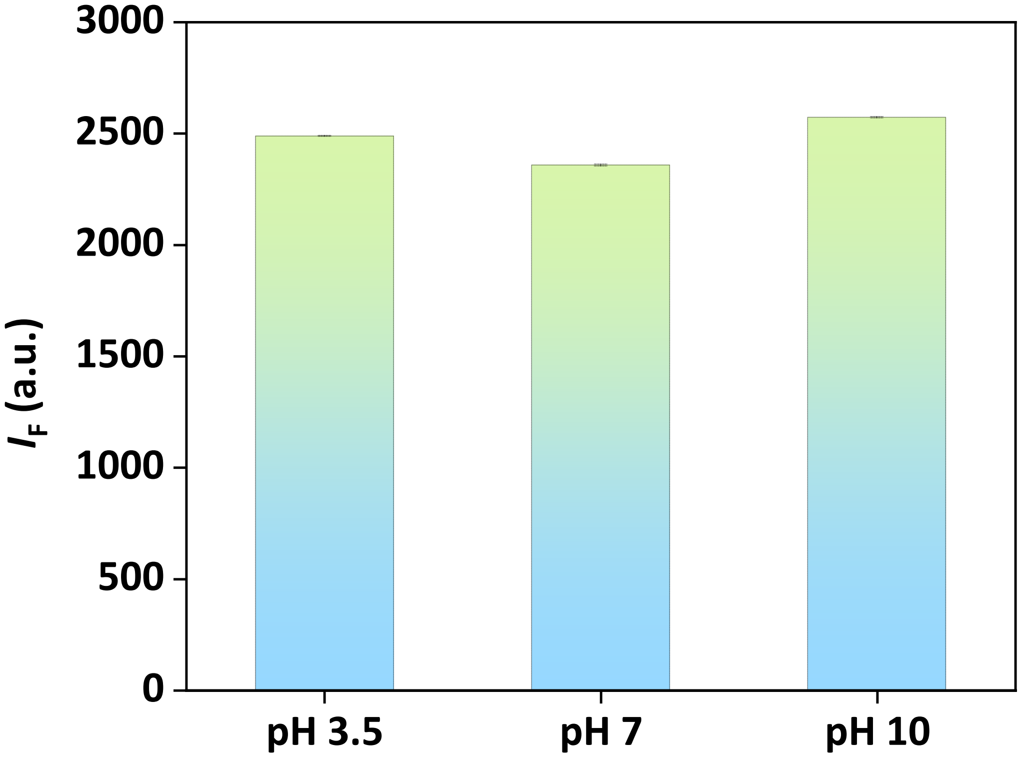

Figure S3. Effect of pH solution on the emission intensity of the Th-F-CMP probe.

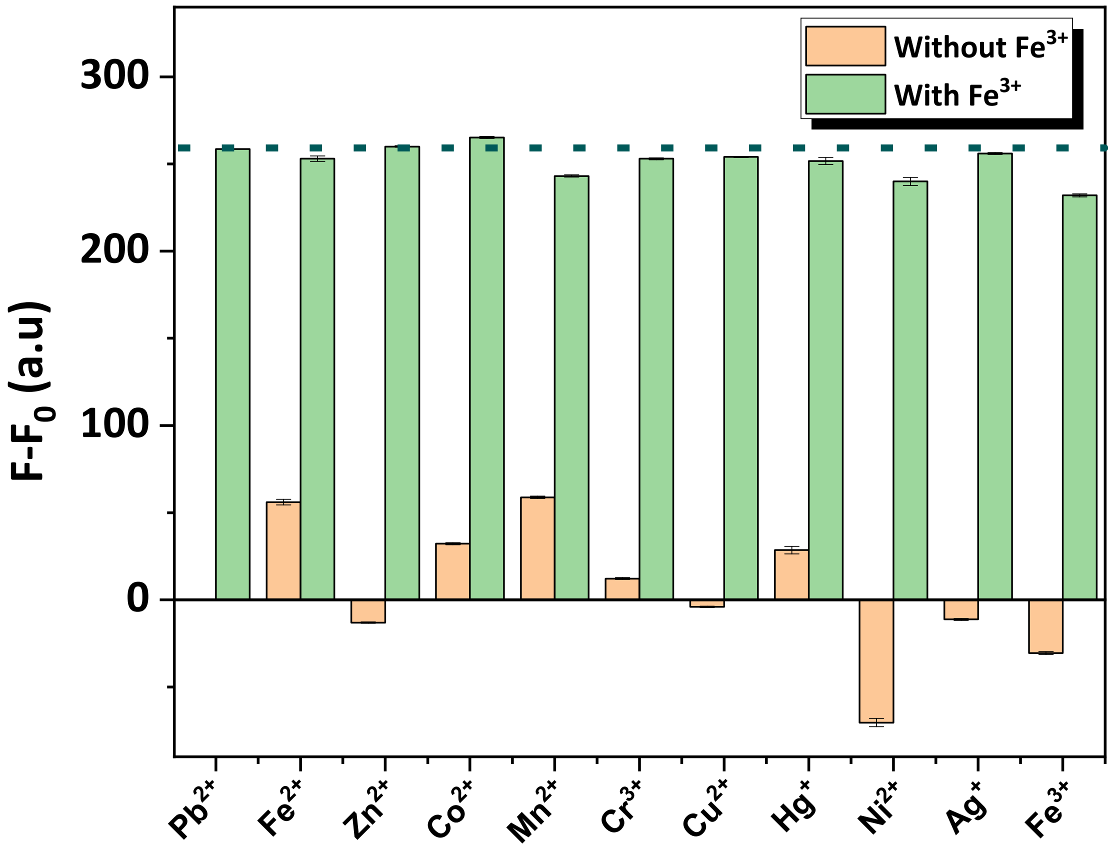

Figure S4. The selectivity of the proposed probe for Fe^{3+} was also evolved in the presence of various other metal ions.
